# Supplementary material for: Patterns of Intron Gain and Loss in Fungi
Source: PLoS Biol. 2004 Nov 30;2(12):e422. doi: 10.1371/journal.pbio.0020422 (PMC532390; doi:10.1371/journal.pbio.0020422)
Supplement: Table S1 — Also available at http://genes.mit.edu/NielsenEtAl/. (4.3 MB ZIP). [file pbio.0020422.st001.zip › NielsenEtAl/html/887.html]

AN8859.1.NCU04118.1.MG06879.1.FG07421.1


```
 CLUSTAL W (1.82) Multiple Sequence Alignments - Introns Inserted


Sequence 1: NCU04118.1	522 aa
Sequence 2: MG06879.1	677 aa
Sequence 3: FG07421.1	486 aa
Sequence 4: AN8859.1	525 aa
Alignment Length: 718 aa
Number Identitical Residues: 209 aa
Alignment Score (without introns) 10660


MG06879.1 	-----------------MKNNSENG-WVVQKFGGTSVGKFPDK~IAEDIVQ~PYLRNNKI
NCU04118.1	-----------MAGPQLLQNDSPNG-WIVQKFGGTSVGKFPDR~IAEDIIR2AYVKDHRL
FG07421.1 	------------MGSLSYNNSPDLG-WVVQKFGGTSVGKFPDK0IAKDIVR2------LL
AN8859.1  	MNGQLQSNPDILSTLKDIPSASHNGNWVVQKFGGTSVGKFAHN~IIDQVVL2PSLSHNKV
          	 ... .:...         . .  *.*:************... * .:::  .      :

MG06879.1 	VVVCSARSSGKKVTGTTSR2LLEVFRRLKSLEI--SIEPTQTELIDEARRIIQDICDDHV
NCU04118.1	VVVCSARSTGKKVTGTTSR2LLEVFKNLKAIAAYASDEHKQDALLKDAKRVMQDICSDHT
FG07421.1 	AVYVKLRGVGAAIC-----~---------------SDEEQQNELVEQARGLMLDICNDHV
AN8859.1  	AVVCSARSSSTKAEGTTNR2LLR------------AARDAENSQSKQYLSFVEAVRLEHV
          	.*  . *. .    .::.                 : .  :    .:   .:  :  :*.

MG06879.1 	AAAETFIRDASRCAMLIKEIQDECLELCEYIVAAKRFHLEVNARSKDRVASFGEKLSCRF
NCU04118.1	AAADKFIQDAELAAIVKEETVKECQLLIEYLEAAKRFHLEVNSRAKDRVISFGEKLSCRF
FG07421.1 	FAAESFIKDQNLRETLIQTVKDECQELVEYIVAAKRFNLEINSRAKDRVISFGEKLSCRF
AN8859.1  	DVVESQINSAELRTQLTSAINAECERVLKVLEAAQTLG-EISARCVDKVISTGEKLSCRL
          	 ..:. *.. .    : .    **  : : : **: :  *:.:*. *:* * *******:

MG06879.1 	MACLLQDR0NVEAQYVDLSDVLSYEG-ARRLDHTFYSDAAAVIRKKILACGDKVPIVTGF
NCU04118.1	MACMLKDR~GVDAEYVDLADVLHLDASIERLDATFYREAAEVIAKKILACENRVPVVTGF
FG07421.1 	MTTLLQDV0GVESEYVDLCDAFHYDA-AGRLDDRFYRTATEAFTRKIAACENRVPVVTGF
AN8859.1  	MAAFLQDR~GVDSEYVDLSEIVDFHISGQGLDQEFYDNLAAALGRKVHACENKVPVITGY
          	*: :*:*  .*:::****.: .  . :   **  **   : .: :*: ** ::**::**:

MG06879.1 	FGNVPGSLLDGDIGRGYTDLCAALIAVGLKAGELQVWKEVDGIFTADPTKVPTARLIGAI
NCU04118.1	FGNVPGGLLDGDVGRGYTDLCASLAAVGLKADELQIWKEVDGIFTADPSKVPTARLVPSI
FG07421.1 	FGNVPGSLIDGDIGRGYTDLCAALLSVGVKAEELQVWKEVDGIFSADPSKVPTARLLSSI
AN8859.1  	FGTIPGGLLD-QIGRGYTDLCAALVAVGVQAGELQVWKEVDGIFTADPRKVPTARLLPAI
          	**.:**.*:* ::*********:* :**::* ***:********:*** *******: :*

MG06879.1 	TPSEAAELTFYGSEVIHHLTMDQVIKADPPIPIRIKNVKNPRGEGTIVVPDAVFAPGKEL
NCU04118.1	TPSEAAELTFYGSEVIHHLTMDQVIKATPPIPIRIKNVKNPRGEGTIVKPDPLLAPDQTL
FG07421.1 	TPSEAAELTFYGSEVIHHLTMDQVIRAEPPIPIRIKNVKNPRGNGTIVVPDRMRSASHQL
AN8859.1  	TPAEAAELTFYGSEVIHPFTMEQVIRAK--IPIRIKNVMNPKGDGTVIFPDSTYELERTT
          	**:************** :**:***:*   ******** **:*:**:: **      :  

MG06879.1 	RRSRP------SELDSVGRKT--PKRPTAVTIKDGISVINVHSNKRSIAHGFFARVFSIL
NCU04118.1	QRHQQNGTCNGTKTNGTGQKSNKPKRPTAVTIKDKISIINIHSNKRSIAHTFLARLFAIL
FG07421.1 	TRGSP------TPKAEAEHRK--PKRPTAVTIKDHISVINVHSNKRSISHGFFARVFSIL
AN8859.1  	PGHDPR-----LFRTRSPSLLQRPKRPTAVTIKHNILVINVHSNKRSLSHGFFAGIFSVL
          	                     . **********. * :**:******::* *:* :*::*

MG06879.1 	DQNQVSVDLISTSEVHVSMAIHAASDNG------------GNFDRARAELGDCGEVSVLH
NCU04118.1	NKHRVSVDLISTSEVHVSVAVHMGNAEM------------TNFDAAVKDLGDCGDVSVLH
FG07421.1 	NHYSISVDLISTSEVHVSMAIHASSQQV------------EAFANAAAELADCGDVSILT
AN8859.1  	DRWKLSIDLISTSEVHVSMALHSERPLLNGVGRDEYQIIDEDLKGALSDLQRYGTVDIIP
          	::  :*:***********:*:*      .. . .. .  .  :  *  :*   * *.:: 

MG06879.1 	DMAILSLVGADMKNMVGVAGKMFSTLGEHNVNIEMISQG1ASEINIS2AALVAFASA1TA
NCU04118.1	GLAILSLVGAEMKNMIGISGKMFSTLGDHRINIEMISQG1ASEINIS~CVIDARDAE~RA
FG07421.1 	DMAILSLVGAEMKNMVGIAGRMFSTLGEHNVNLEMISQG1ASEINIS~CVISARDAT~RA
AN8859.1  	GMAILSLVGKQMKNMVGVAGRMFSTLGENNVNIEMISQG1ASEINIS~CVIEERDAD~RA
          	.:******* :****:*::*:******::.:*:****** ******* ..:    :   *

MG06879.1 	MTLASIALPNWISYNITSIKGHSITRNIGLHKTCSSGIDPFSDSPTCRPFPIEREDCAGD
NCU04118.1	MNILHTNLFTFLE-----------------------------------------------
FG07421.1 	MNVLHTHLFTFLE-----------------------------------------------
AN8859.1  	LNIIHTSLFTFLD-----------------------------------------------
          	:.:    * .::.                                               

MG06879.1 	ERGFCSIWRTTGFLMSLAIVVELATLVGFLVIMAGGKMKRVTGWKILSCLLVSIATLLLM
NCU04118.1	------------------------------------------------------------
FG07421.1 	------------------------------------------------------------
AN8859.1  	------------------------------------------------------------
          	                                                            

MG06879.1 	SMGTV0AYIFDNDDKFSVPGWSLDKSWIMTTVSAAFCLLCAVVFPVSAHFLPAEGDYEFL
NCU04118.1	-----~------------------------------------------------------
FG07421.1 	-----~------------------------------------------------------
AN8859.1  	-----~------------------------------------------------------
          	                                                            

MG06879.1 	RDPVHA
NCU04118.1	------
FG07421.1 	------
AN8859.1  	------
          	
```
